# Load Monitoring and Its Relationship with Healthcare in Sports

**DOI:** 10.3390/healthcare11162330

**Published:** 2023-08-18

**Authors:** Rafael Oliveira, João Paulo Brito

**Affiliations:** 1 Sports Science School of Rio Maior, Polytechnic Institute of Santarém, 2040-413 Rio Maior, Portugal; jbrito@esdrm.ipsantarem.pt; 2 Life Quality Research Centre, 2040-413 Rio Maior, Portugal; 3 Research Centre in Sport Sciences, Health Sciences and Human Development, 5001-801 Vila Real, Portugal

Load monitoring consists of training/match demand quantification as well as wellness and readiness to maximize the likelihood of optimal athletic performance [1]. The literature divides load into two dimensions: internal and external. Internal load is associated with psychophysiological demands that can be objectively and subjectively measured (e.g., heart rate and rating of perceived exertion, respectively) [2,3]. External load is associated with mechanical/locomotor demands, usually collected by global positioning systems, global navigation satellite systems, local positioning systems, and inertial measurement units that belong to micro-electro-mechanical systems (which provide a combination of 3D accelerometers, 3D gyroscopes, and 3D magnetometers). Despite different technologies, they provide external load measures, such as distances covered at various running speeds, accelerations, decelerations, player load, and others [2,3,4,5].

Indeed, there are other types of wearable technology that were considered to be among the top worldwide fitness trends in 2016 and 2017 [6]. Such technology (i.e., smartwatches and mobiles) allows for the quantification of different physical variables such as step counts, metabolic work, or power [7]. 

Another relevant dimension to monitor is the wellness/well-being of athletes, which is regularly collected by questionnaires that include different categories such as fatigue, quality of sleep, muscle soreness, mood, and stress [8,9]. For instance, a systematic review showed several relationships between wellness and training load measures that ranged from no association to a very large association [10].

The monitoring of different dimensions is useful in sports to optimize training adaptation, which can consequently improve performance and reduce injury risk [11]. Still, inappropriate load management can be a significant risk factor for acute illness and overtraining syndrome [12]. Therefore, all quantification can contribute towards better healthcare for recreational or elite sport athletes. However, there are few research papers that combine load monitoring and its relationship with healthcare in sports.

The present Special Issue contributed to the field with 35 articles. Of those, 16 articles involved only soccer athletes. For instance, one study was a systematic review that summarized studies about external and internal training load monitoring to provide range values for the main measures in young male soccer players [13]. Another study compared the external load between official and friendly matches and between the first and second halves of professional soccer players [14], while another analyzed differences among playing positions: whether playing home/away matches and if playing in the first or second part of the championship influence the external load of amateur soccer [15]. Moreover, external load was compared between starters and non-starters [16] and among the playing positions [17] of professional soccer players based on different parts of a full season. A sub-analysis of a specific type of training exercise (i.e., small-sided games) was performed to analyze the between-session and within-player variability of heart rates and external load of young male soccer players [18]. While the previous literature included male athletes, the analysis of female professional soccer players was also conducted by quantifying external and internal load as well as the wellness profile of a typical microcycle [19]. Another study tested objective and subjective external and internal loads in primary education students [20].

Other investigations included the analysis of physical fitness and competitive performance in different age categories (8.0–9.9, 10.0–11.9, and 12.0–13.9 years) [21] and in professional soccer [22]. In this regard, different intervention protocols were applied to professional soccer players [23,24] and archers [25] to improve several fitness characteristics. In the same way, fitness and technical skill were compared among young soccer players [26]. Additionally, the relationship between different inter-limb jumping asymmetries and performance measures in male senior and professional soccer players was analyzed [27]. Furthermore, the effectiveness of different training programs on the reactive strength index was compared in a systematic review with a meta-analysis [28], isotonic and isometric exercise interventions were reviewed to analyze the strength and flexibility of the hamstring muscles [29], and a dose-response meta-analysis was conducted to assess the velocity loss effects on strength development and related training efficiency [30]. In fact, other authors analyzed different sports training protocols. For instance, aquatic and bicycling training was applied to improve leg function and range of motion in the intermediate stage of rehabilitation in amateur athletes that underwent meniscal allograft transplantation [31].

Other negative psychological variables, such as stress, injury, anxiety, and depression in adult soccer players, were also analyzed [32]. In this regard, one study applied a mindfulness program to address levels of impulsivity, mood, and pre-competition anxiety in samples of athletics, tennis, swimming, basketball, handball, volleyball, and soccer athletes [33]. Coping strategies were another topic analyzed in professional soccer during Ramadan fasting [34], as well as in other sports than soccer, such as handball, martial arts, rugby, basketball, athletics, aerobic and artistic gymnastics, volleyball, tennis, and swimming during the COVID-19 pandemic [35]. Lastly, the personality and resilience of competitive drivers were another topic of research [36].

In basketball, one study compared the redox, hormonal, metabolic, and lipid profiles between adult male and female athletes and sedentary controls [37], while others characterized the salivary proteome and metabolome of highly trained female and male young basketball players [38].

Body composition and rapid weight loss were another topic of analysis for combat athletes [39]. Moreover, not only body composition but also physiology and morphology were compared between male and female Olympic-distance triathletes [40,41]. Another study analyzed the effects of low-intensity aerobic training combined with blood flow restriction on body composition, physical fitness, and vascular responses in recreational runners [42]. An analysis of the vitamin D receptor (VDR), the rs2228570 polymorphism, and its effect on elite athletes’ performance was also compared between track and field athletes with non-athletes (controls with a physical activity record) [43].

In tennis, high-intensity interval training effects in athletes with and without cognitive load were analyzed on accuracy, critical flicker fusion threshold, and rating of perceived exertion [44].

In Olympic weightlifting, a comparison of the fatigue prompted by the “Clean and Jerk”, and “the Snatch” and their derivative exercises among male and female participants was performed [45].

A case study about the influence of swimming training on an athlete with active Chron’s disease, where scarce research exists, was conducted [46]. Finally, another case study analyzed four athletes who participated in a 768 km ultra-trail race for 11 days to address bone turnover alterations [47].

This Special Issue provides relevant information to update the state of the art in this field. It addresses several sports, including young, professional, recreational, male, and female athletes. Moreover, it addresses some gaps in the literature (e.g., Chron’s disease, Olympic weightlifting, or velocity speed loss). Notwithstanding, this Special Issue, along with its included studies, contributes information to improve load monitoring (of training and competition) and healthcare through direct or indirect research.
